# Travel ban effects on SARS-CoV-2 transmission lineages in the UAE as inferred by genomic epidemiology

**DOI:** 10.1371/journal.pone.0264682

**Published:** 2022-03-02

**Authors:** Andreas Henschel, Samuel F. Feng, Rifat A. Hamoudi, Gihan Daw Elbait, Ernesto Damiani, Fathimathuz Waasia, Guan K. Tay, Bassam H. Mahboub, Maimunah Hemayet Uddin, Juan Acuna, Eman Alefishat, Rabih Halwani, Herbert F. Jelinek, Farah Mustafa, Nawal Alkaabi, Habiba S. Alsafar, on behalf of the UAE COVID-19 Collaborative Partnership

**Affiliations:** 1 Department of Electrical Engineering and Computer Science, Khalifa University, Abu Dhabi, United Arab Emirates; 2 Center for Biotechnology, Khalifa University of Science and Technology, Abu Dhabi, United Arab Emirates; 3 Department of Mathematics, Khalifa University of Science and Technology, Abu Dhabi, UAE United Arab Emirates; 4 Sharjah Institute for Medical Research, University of Sharjah, Sharjah, United Arab Emirates; 5 Department of Clinical Sciences, College of Medicine, University of Sharjah, Sharjah, United Arab Emirates; 6 Division of Surgery and Interventional Science, UCL, London, United Kingdom; 7 Center for Cyber Physical Systems, Khalifa University of Science and Technology, Abu Dhabi, United Arab Emirates; 8 Division of Psychiatry, Faculty of Health and Medical Sciences, The University of Western Australia, Crawley, Western Australia, Australia; 9 School of Medical and Health Sciences, Edith Cowan University, Joondalup, Western Australia, Australia; 10 College of Medicine, University of Sharjah, Sharjah, United Arab Emirates; 11 Department of Pulmonary Medicine, Rashid Hospital, Dubai Health Authority, Dubai, United Arab Emirates; 12 Department of Pediatric Infectious Disease, Sheikh Khalifa Medical City, Abu Dhabi, United Arab Emirates; 13 Department of Epidemiology and Population Health, College of Medicine and Health Sciences, Khalifa University of Science and Technology, Abu Dhabi, United Arab Emirates; 14 Office of Academic Affairs, Directorate of Research and Development, SEHA, Abu Dhabi, United Arab Emirates; 15 Department of Pharmacology, College of Medicine and Health Sciences, Khalifa University of Science and Technology, Abu Dhabi, United Arab Emirates; 16 Department Biopharmaceutics and Clinical Pharmacy, Faculty of Pharmacy, The University of Jordan, Amman, Jordan; 17 Health Engineering Innovation Center, Khalifa University of Science and Technology, Abu Dhabi, United Arab Emirates; 18 Department of Biomedical Engineering, College of Engineering, Khalifa University of Science and Technology, Abu Dhabi, United Arab Emirates; 19 Department of Biochemistry and Molecular Biology College of Medicine and Health Sciences, United Arab Emirates University, Al Ain, United Arab Emirates (UAE); 20 Department of Genetics and Molecular Biology, College of Medicine and Health Sciences, Khalifa University of Science and Technology, Abu Dhabi, United Arab Emirates; Instituto Adolfo Lutz, BRAZIL

## Abstract

Global and local whole genome sequencing of SARS-CoV-2 enables the tracing of domestic and international transmissions. We sequenced Viral RNA from 37 sampled Covid-19 patients with RT-PCR-confirmed infections across the UAE and developed time-resolved phylogenies with 69 local and 3,894 global genome sequences. Furthermore, we investigated specific clades associated with the UAE cohort and, their global diversity, introduction events and inferred domestic and international virus transmissions between January and June 2020. The study comprehensively characterized the genomic aspects of the virus and its spread within the UAE and identified that the prevalence shift of the D614G mutation was due to the later introductions of the G-variant associated with international travel, rather than higher local transmissibility. For clades spanning different emirates, the most recent common ancestors pre-date domestic travel bans. In conclusion, we observe a steep and sustained decline of international transmissions immediately following the introduction of international travel restrictions.

## Introduction

COVID-19 is an emerging disease caused by a novel beta coronavirus, SARS-CoV-2 [1]. The first cases of the disease were described in patients from Wuhan, China [2]. It has affected nearly all countries of the world, causing 900,000 deaths and infecting nearly 30 million people by early October 2020 [3]. Understanding the pandemic at the molecular level through viral genome sequencing is paramount for tracing the epidemic spread, and also for diagnostics as well as vaccine and antiviral drug development since regional differences in viral sequences may affect both the drug and vaccine efficacy. The first COVID-19 case in the United Arab Emirates (UAE) was reported on the 29^th^ of January 2020. As of September 2020, the UAE has registered 74,454 cases of SARS-CoV-2 (around 48,000 during this study period) [4].

Genomic epidemiology aims to trace transmission lineages of a pathogen to characterize the spread of an epidemic or pandemic using observed mutations in a selection of sampled genomes over time and space. Fauver et al. [5] established a phylogeographic method to trace the transmission of lineages and demonstrated the effectiveness of this approach for the spread of SARS-CoV-2 across the USA. The computational framework has been previously applied to several epidemics, such as the one that was caused by the Zika virus [6]. This approach is based on the spatio-temporal construction of phylogenies from full genomes of different strains of the same pathogen over time and space. Using a range of metadata, in particular geographic locations and sampling times, the internal nodes of the phylogenetic trees—i.e., hypothetical ancestors—can be characterized and annotated, including the time and location of their existence as well as their most likely genomic sequence. This helps to trace the most likely scenario of the spread of the pathogen. Software toolkits that are available for this type of probabilistic evolutionary analyses include TreeTime and the Nextstrain/Augur tool suite [6, 7]. A similar effort is presented in Pybus et al. [8], where the authors conducted a large-scale genomic epidemiology analysis with more than 20,000 SARS-CoV-2 genome sequences and postulated that the virus entered the United Kingdom (UK) on more than 1,356 independent occasions [9, 10]. Furthermore, the analysis of epidemic spread including international introductions using viral genomes have been conducted for a range of countries, including Brazil [11], India [12] and Bangladesh [13].

Comparing the results of our genomic epidemiology analysis to local and international travel restrictions provided insights into the efficacy of interventions. The UAE has approximately 9.9 million inhabitants in seven emirates. The two major cities of the UAE are Abu Dhabi (1.4 million inhabitants) and Dubai (3.3 million inhabitants), which are around 140 km apart. The two cities are capitals of their respective emirates, and each is a major international passenger and cargo traffic hub. The Dubai International Airport served over 88 million passengers in 2019 [14]. The country’s air traffic volume totals to roughly 740 departures per day under normal circumstances, according to the International Civil Aviation Organization (ICAO) [15]. Large road-based traffic volumes flow between the two cities. However, land-based international traffic is comparatively low with only a few border posts neighboring Saudi Arabia and Oman. From an epidemiological perspective, it seems plausible that the nearly complete shut-down of UAE airports led to changes in transmission patterns of SARS-CoV-2. This strict and expensive intervention requires justification and therefore necessitates exact efficiency assessment. This study sought to provide evidence through genomic epidemiology, that airport closures would lead to a substantial and sustained decline of international transmissions. We emphasized the differences, similarities and potential regional transmission lineages between Dubai and Abu Dhabi during the COVID-19 pandemic as these interventions also affected the travel between the two cities. To understand the regional transmissions, spatially representative sampling across the two UAE metropoles was also applied.

## Results

We successfully extracted RNA from 71 COVID-19 patients between April 3rd-July 1st, 2020. Of these, 37 samples, 32 from Abu Dhabi and 5 from Dubai, showed FASTQ Phred quality scores above 30 for all trimmed reads, as demonstrated by FASTQC. The sequences also exhibited at least a minimum coverage of 30X (up to 2,745-fold, on average 435-fold) across the entire reference genome Wuhan-Hu-1 (GenBank Identifier MN908947). Due to the ultra-deep sequencing, the GATK genotype quality score (GQ) was maximum (99) and root mean square mapping quality (MQ) was > 59.9 for throughout the entire sequences in the selected samples, necessitating no further variant filtration or low quality masking. Whole genomes for the 37 samples were assembled using reference-based genome assembly.

### Sample demographics

UAE is a regional hub for people from 196 different nationalities. Therefore, it is no surprise that of the 37 patient isolates analyzed in this study (aged 3–71 years; 76% males), only 19% were Emiratis primarily from the emirates of Abu Dhabi (86%) and Dubai (11%) (S1 Table). The remaining individuals represented eight other nationalities, including 35% from India, 16% Egypt, 8% Pakistan, 5% Bangladesh/Nepal/Philippine, and 3% Syria/Sudan (S1 Table).

### Phylogenetic analysis

**Fig 1:**
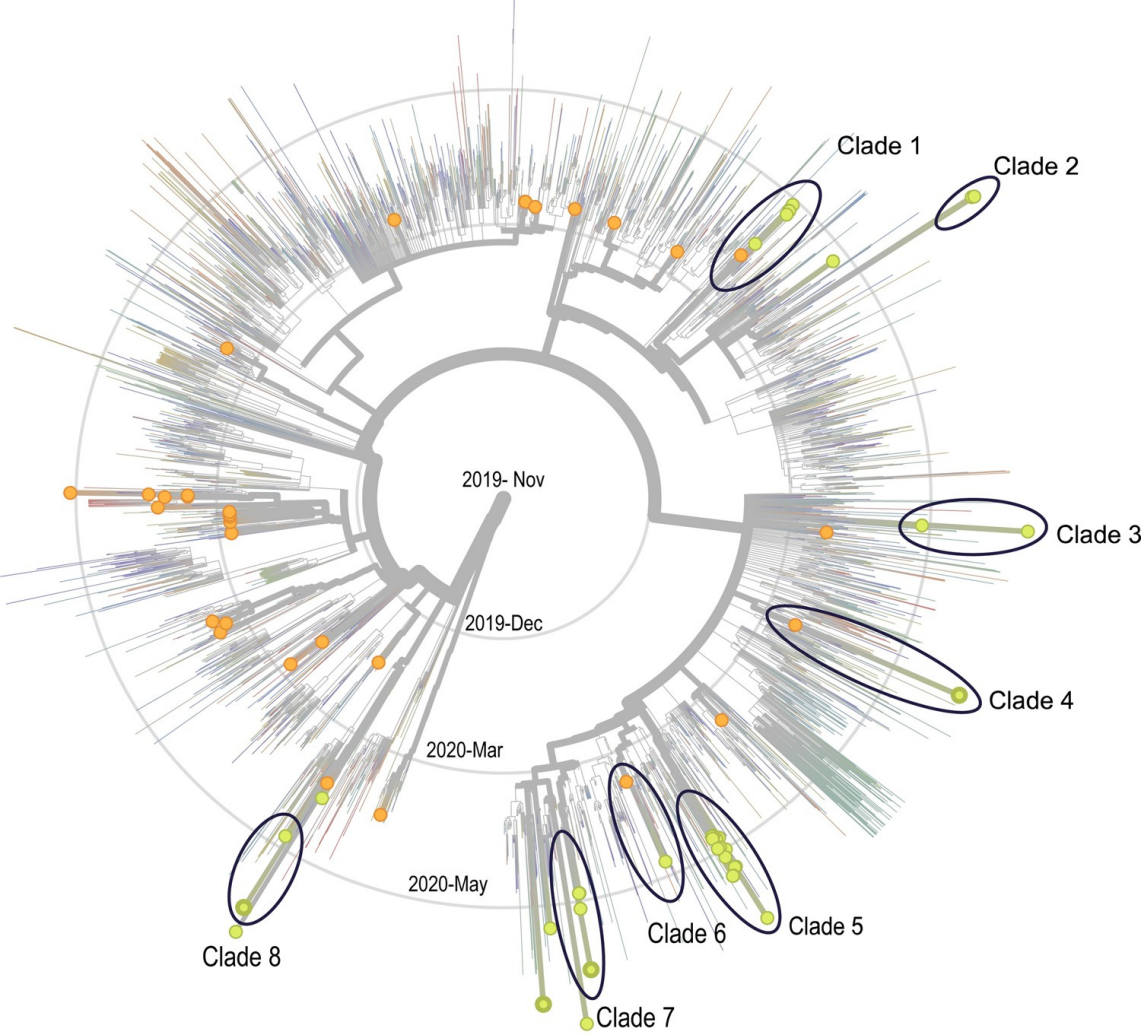
Phylogenetic tree of the 69 UAE genome sequences in the context of global data. A subsampled tree displaying 8 Abu Dhabi clades with multiple UAE descendants in ovals. Samples collected for this study are from Abu Dhabi (green) and Dubai (green with bold outline). Samples from Tayoun et al. are shown in orange.

We constructed a phylogenetic tree, which guarantees inclusion of all strains that are similar to UAE sequences from the pool of high-quality strains published in GISAID for the relevant time span. Since conventional random sub-sampling does not provide such a guarantee and thus could miss out on “smoking guns” for international introductions, we argue that our approach is superior for the purpose of UAE import detection. The all-encompassing phylogeny with 3,894 GISAID (including 721 sequences from k-nearest neighbor search, kNN) and 69 local sequences provided a comprehensive, global contextualization of the UAE strains. These strains are broad and diverse (Fig 1), suggesting multiple introductions into the country (see next section for more details). This was verified by analysis of the phylogeny shown in Fig 1, which revealed that the 37 UAE samples from our laboratory fell into clades with 12 to14 independent introductions into the UAE. They continued to occupy diverse clades spanning PANGOLIN lineages A, B.1, B.1.1 and B.1.5, as well as B.1 subclades. Interestingly, 86.5% of the samples (32/37) were observed to belong to lineages B.1 (n = 7) and B.1.1 (n = 25) (S2 Table).

### SARS-CoV-2 sequence similarities and intra-host variation

We generated a 17,044x69 distance matrix as part of the kNN calculation, comparing all redundancy and quality filtered GISAID sequences against all 69 UAE (37 from our lab and 32 already present in GISAID, submitted by Tayoun et al. [16]). The collective kNN search (k = 25) yielded 721 sequences with most matches from the UK (163), United States of America (USA) (78), Australia (55), China (43), Austria (32), India (19), Belgium (18), Singapore (16), Portugal (15) and Switzerland (13).

**Fig 2:**
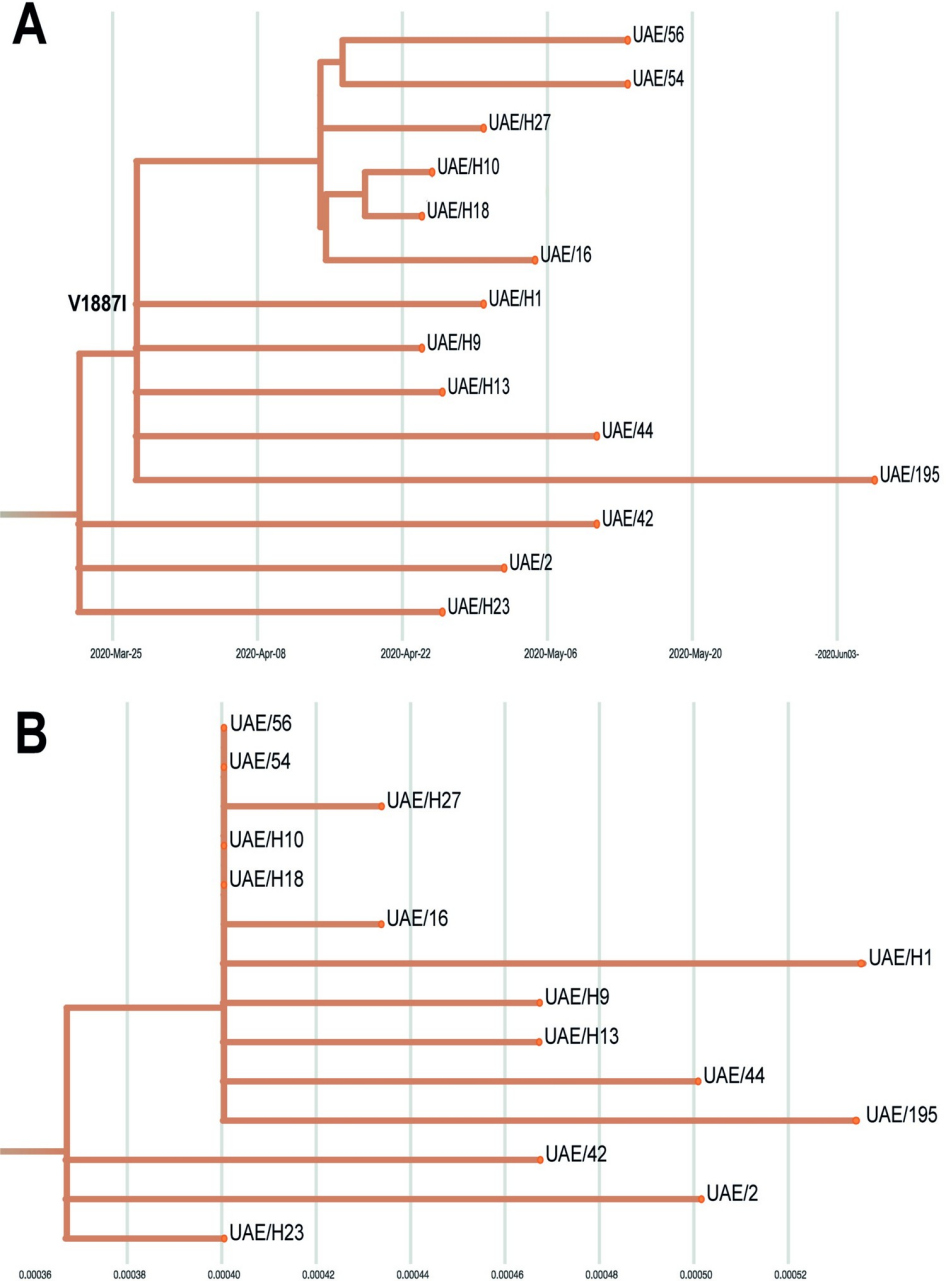
Abu Dhabi’s largest cluster (clade 5). A. Time tree for UAE clade 5. B. Divergence tree for the same clade. We identify four identical UAE strains that appear along a vertical line (with 2 mutations with respect to the clade ancestor).

We identify four genomes (H18, 56, H10, 54) identical in terms of consensus, that are also unique to Abu Dhabi (Fig 2B). These are potential indicators for domestic hotspots and/or superspreading events. In addition to these four identical genomes, another identical pair of genomes was observed in samples 31B and 21R that were isolated from patients with Indian and Bangladeshi national backgrounds, respectively, once again suggesting a domestic transmission/hotspot (S2A Fig). Further to consensus sequences, we also report within-host diversity in order to elucidate how SARS-Cov-2 is mutating within a patient. This approach is enabled by the deep sequencing protocol (16-2641-fold coverage on polymorphic sites). Of the 36 samples, 18 variants contain in total 68 polymorphic variant calls. After filtering minor allele frequency (MAF) with > 5%, we retain 12 intra-host Single Nucleotide Variants (iSNVs) from 3 different samples (S4 Table). In particular, iSNVs in H18 were reproduced in a second run with depths between 943–1246. Remarkably, in the unfiltered callset, few loci appear in multiple samples. E.g. loci 28881–28883, 14408 (7 samples), locus 241 (6 samples) and locus 23403 (3 samples). The latter three loci have also been shown to be frequently polymorphic in a UK based study [23]. Possible explanations for iSNVs pattern similarity include similar convergent evolution in different hosts or co-transmission [17].

### Detection and characterization of SARS-CoV-2 transmissions

**Fig 3:**
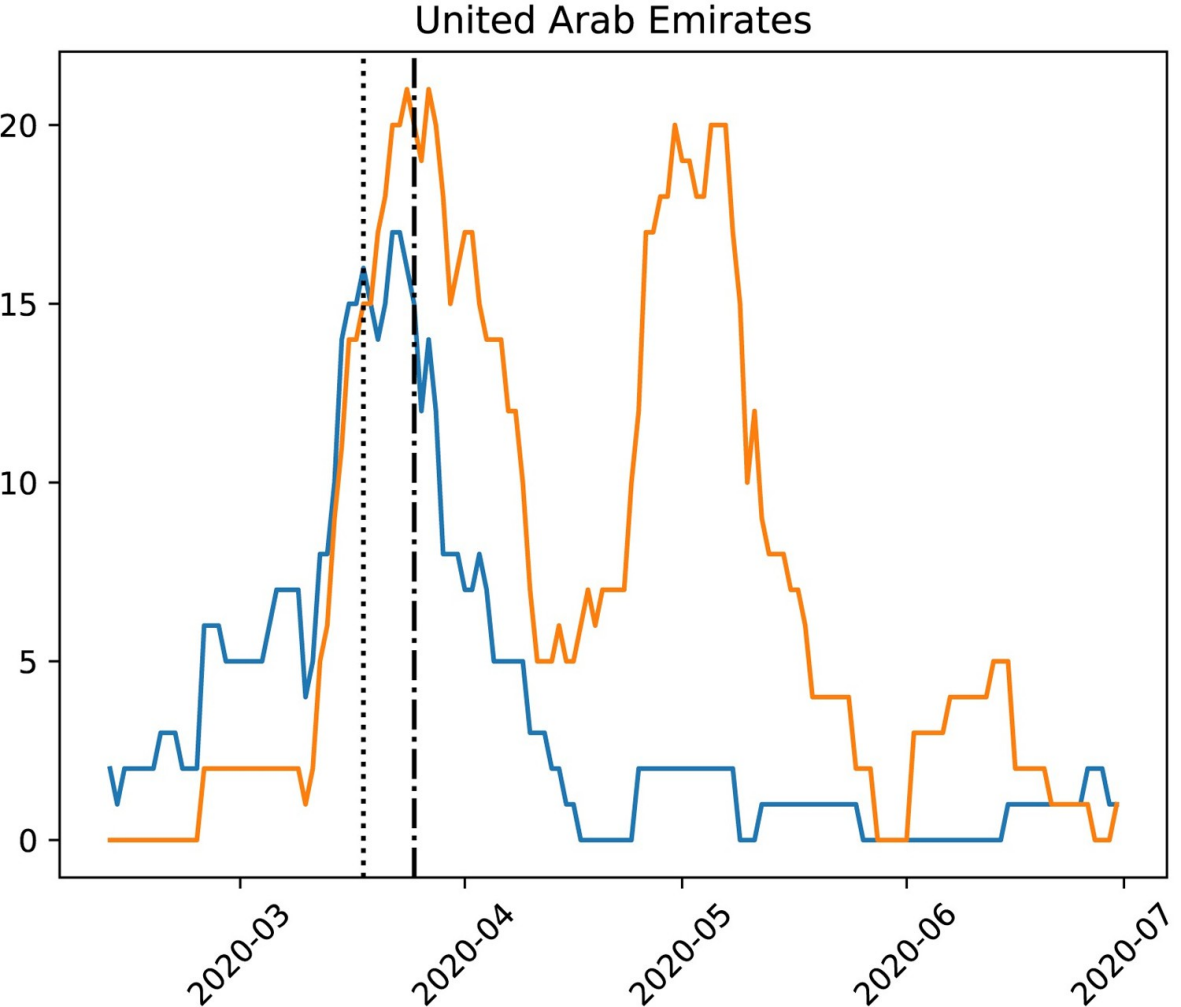
Comparison of international and domestic transmissions over time. The dotted and dash-dotted vertical lines mark the time of the travel ban to/from China and globally, on March 18 and March 25, 2020, respectively. A nearly instantaneous steep drop in international transmissions (blue) can be observed. We also observe that domestic transmissions (orange) continued to occur.

Transmissions from international travelers entering the UAE decreased over time, while the same was not true for domestic transmissions by the local population. A substantial part of reductions in international transmissions appears to be attributable to international travel restrictions, as the timing of the international transmission drop coincided exactly with international travel ban restrictions. Fig 3 shows a steep drop in inferred international transmissions after the introduction of a complete international travel ban on March 25, 2020. A variety of domestic interventions, such as the closure of schools, offices, and a nationwide disinfection program, including a nightly curfew, were also implemented throughout the month of March [18]. The slight rise in cases observed following the travel ban (Fig 3) probably is due to the international travelers already in the UAE who became positive due to the incubation period between infection and symptom appearance which could range between (4–14) days.

On the other hand, most infections in April and May can be attributed to a steep rise in domestic transmissions. We note that this increase in domestic transmissions may correspond to the Islamic month of Ramadan (April 23 –May 23), traditionally marked by communal celebrations and gatherings for meals. For decision makers in the government, it is thus informative to see that global travel restrictions appeared to be a successful strategy, whereas a strong focus on domestic intervention must also be implemented to curb the local spread. This characteristic seems unsurprising given the relatively small size of the country. However, a similar analysis for 30 countries shows that no other country except Singapore, which is of similar size, exhibited such a sharp drop of international transmissions upon banning international travel (S3 Fig).

Our analysis identified transmissions across the Emirates, since the most recent common ancestor, MRCA (and all relevant descendants) of clades comprising Dubai and Abu Dhabi samples are estimated to be of UAE origin with a confidence of 66% or higher (see S2 Fig).

The times of the most recent common ancestors (TMRCA) for clades comprising Dubai and Abu Dhabi samples are estimated to be early January and early March. It is therefore highly likely that transmission happened before the inter-Emirate travel ban on 19-03-2020 (https://wam.ae/en/details/1395302831731). No TMRCAs for Dubai/Abu Dhabi clades were observed after that date, indicating the effectiveness of the Emirate travel ban, and suggesting that phylogenetic analysis can be used to assess the effectiveness of such interventions for preventing the spread of COVID-19.

### Prevalence shift of D614G variants

**Fig 4:**
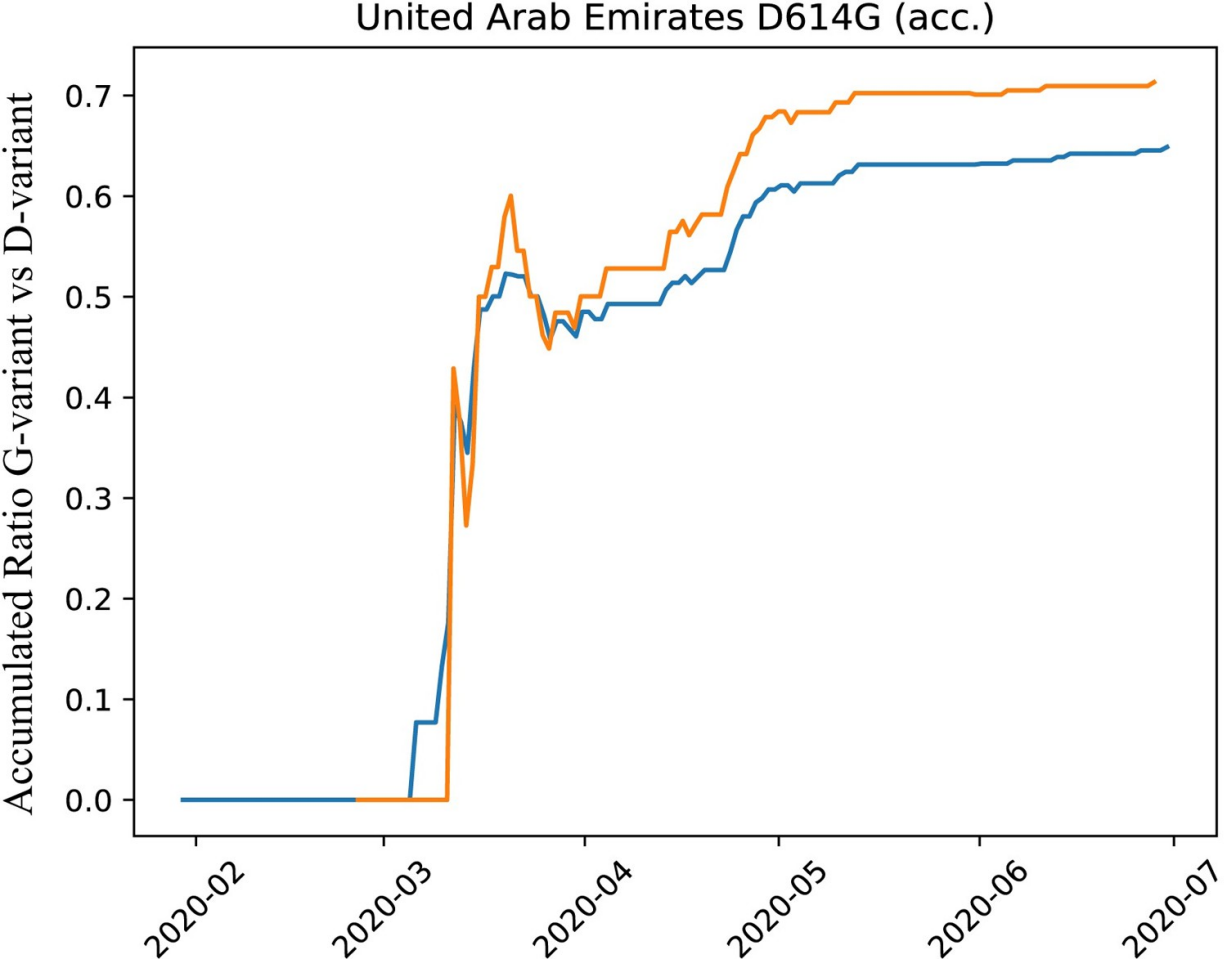
A shift of prevalence of strains with D614G mutations. We measure the accumulated fraction of G-variants vs D-variants over time, while distinguishing international (blue) and domestic (orange) transmissions. The y-axis holds the total accumulated fraction of the G-variant. If the G-variant was substantially more transmissable, we would expect the domestic G/D ratio to clearly and increasingly dominate the international G/D ratio, in particular in light of the stronger domestic epidemiology after April 2020.

We also observed a shift of prevalence for strains with D614G mutations in the viral Spike (S) protein. A potential explanation is increased with increased infectivity [19], although this has been disputed [20]. Fig 4 shows the gradual increase of G-variants to an accumulated count of 65% and can be attributed to the change of import origins as initially (until early March 2020), only D-variants were introduced from Asia, in particular from China. During the study period, a rise of G-variants from predominantly European origins, can be observed. It follows also from Fig 4 that this relative increase is better explained with increased imports and not attributable to higher transmissibility in the UAE.

### Characteristic mutations

The comparative sequence analysis identified mutations–both on the amino acid and the nucleotide level–that are either of high genomic diversity (entropy) or unique to the UAE. S3 Table lists three amino acid mutations (almost) unique to the UAE and the three amino acid mutations with highest entropy in the UAE. In addition to the above-mentioned D614G mutation, we observe four cases of a nearby E583D mutation, that could be of diagnostic or clinical relevance (transmissibility and severity). We discovered one case of Q613H, directly adjacent to position 614 in S, almost unique to the UAE. The only other cases with this mutation are rather remote with respect to both phylogeny and geography (found in Japan) and likely to be convergently evolved. Apart from the S protein, an Abu Dhabi specific, monophyletic amino acid mutation in ORF1a (V1887I), which preserves physico-chemical properties (both are aliphatic, BLOSUM62 score of 3) was detected. Remarkably, the mutation can be dated to around March 25, 2020 (see Fig 2A), as it is present in 11 out of the 14 samples which form the largest UAE clade (clade 5 in Fig 1).

## Conclusions

Several epidemiological analyses for COVID-19 have identified multiple introductions of the SARS-CoV-2 virus into several countries, including the UAE, UK, USA, and Brazil [8, 10, 11, 16]. Thus, from a national perspective, there is not a single patient that is ancestral to all infections for a specific country. In this study, we constructed a phylogenetic tree that shows SARS-CoV-2 strains present in the UAE during early on-set of the pandemic, comprehensively contextualized with international samples. The annotated tree displays the diversity and provides insights into domestic transmission and international introduction patterns. We show that even though multiple international introductions happened (Fig 1), import events declined drastically in the UAE during the period of international travel restrictions, while domestic transmissions persisted (Fig 3). Moreover, the decline was sustained over several months (April-June 2020; Fig 3), while air travel was gradually reopened during which the pandemic was still increasing worldwide. However, although the later sequences are’t new introductions, they still carry a high signal of entropy and diversity in terms of phylogenetic distance (Fig 1 and S3 Table). Thus, even if milder variants arise (for example, the ORF8 382-nucleotide deletion mutant [21]), their impact will likely be limited due to the observed diversity sustained throughout the sampling period of five months.

We also conclude from our results that the Inter-Emirates travel ban was efficient. Moreover, we report a prevalence shift in D614G, but attribute it to change of import origins rather than higher transmissibility of the G-variant. Finally, we identify identical sequences including identical heterozygosity loci indicating co-infection or co-transmission and UAE specific mutations in SARS-CoV-2.

## Discussion

Aviation and tourism are substantial components of the UAE’s GDP (13%) [22]. The shut-down of airports has therefore huge economic consequences and needs to be coordinated with necessary public health interventions for Covid-19. Had we observed a high level of international transmissions in the time after the travel ban, the efficacy of the measure could be questioned. Our analysis of viral genomes indeed corroborates the initial hypothesis of efficient international travel bans for the case of the UAE. However, travel bans do not guarantee complete elimination of international virus introductions. For example, the USA introduced a partial travel ban against China on January 31, 2020, yet our analysis shows many international transmissions in February and March (S3 Fig).

During the period of this longitudinal study (March-June, 2020), international cargo flights continued and aviation passenger volume gradually increased under strict conditions. These conditions included a reduction of flights from six daily UAE departures (early April, according to ICAO) to 60 departures (late June). Travelers and staff were tested for COVID-19 and/or quarantined as well as airports and airlines conducting safety and hygiene measures, such as social distancing and compulsory wearing of masks. The current results show that genomic epidemiology is a suitable methodology to gauge the efficacy of travel restrictions and can work as a quality control for air travel reopening measures world-wide.

Our analysis has been designed so that domestic and international transmission lineages are distinguishable from each other and are robust as phylogeny reconstructions with different subsampling strategies have reproduced the strong decline signal of international transmissions during the international lock-down. We observe similar effects in a number of countries (S3 Fig). Of course, the absence of numerical evidence for international and regional transmissions after the respective travel bans is not necessarily evidence for absolute absence, given the sample size of 69 UAE sequences. However, we argue, that if rampant international and regional transmissions were present after April 2020, it is unlikely that they would have remained entirely undetected with our methods, especially with our highly sensitive kNN subsampling technique that, to our knowledge, has not yet been used in genetic epidemiological studies for SARS-CoV-2.

Exact origin detection of international lineages using the presented tools has been less reproducible since confidence calculations for countries of origin vary substantially depending upon the amount of subsampled sequences from various countries. Our algorithm attempts to mitigate country contribution imbalance, and we simplify geographic origin detection to domestic vs international. With a comparatively slowly evolving virus and remarkably high sequence identity (up to 100%) of strains, the resolution of polytomies remains a substantial challenge.

The observed signal of potential co-transmission of a major and a minor strain is remarkable (Fig 2A). The sample with the highest number of iSNVs (H18) contains prominent iSNVs (notably loci 241, 14408 and 23403) that were also observed in multiple local as well as UK samples ([23], Fig 5 therein). None of our samples had a travel history to the UK. A likely explanation for the observed intra-host diversity similarities in the UK and the UAE are general evolutionary constraints, permitting viable mutations preferably on few locations. Alternatively, co-transmissions as described in [17] could explain the observed intra-host variation patterns.

Our method was limited by differences in sampling strategies (e.g., randomization, spatially and temporally representative sampling) in various countries. Therefore, we kept the focus on the UAE where the level of sample randomization was known.

## Materials and methods

### Ethics

This study was approved by the Abu Dhabi Health COVID-19 Research Ethics Committee (DOH/DQD/2020/538), SEHA Research Ethics committee (SEHA-IRB-005), and Dubai Scientific Research Ethics Committee (DSREC-04/2020_09)

### Sample collection

In the early stages of the COVID-19 pandemic, the UAE government convened a national COVID-19 pandemic response committee to oversee the operationalization of programs and workflows that were required to control of the SARS-CoV-2 pathogen. This national committee decided that patients and those suspected to have been in contact with infected individuals would be referred to only one medical center in Abu Dhabi (Sheikh Khalifa Medical City) and only one medical center in Dubai (Rashid Hospital).

We selected a convenience sample of 628 COVID-19 patients from these two partnering hospitals in Abu Dhabi and Dubai. Samples were collected as individuals presented at these sites either for PCR testing or were showing symptoms consistent with COVID-19.

Within this sample of diverse patients, we selected 71 patients (37 samples) that provided diverse demographic characteristics similar to that of the UAE. Patients selected were not from the same household, house, or immediate local neighborhood. The characteristics of the chosen samples are provided in S1 Table.

Thirty-six nasopharyngeal swabs and one lung lavage sample were collected from clinically confirmed SARS-CoV-2 positive patients admitted to Sheikh Khalifa Medical City (SKMC)-Abu Dhabi and Rashed hospital in Dubai during the period of April-July 2020. The collection of swabs was performed in UTM™, COPAN’s media collection tubes, in accordance with the protocol approved by The Department of Health, Abu Dhabi.

### RNA isolation

The collected samples were processed for viral RNA extraction using EZ1 Virus Mini Kit v2.0 according to manufacturer recommendations in EZ1® Advanced automated system by Qiagen which was carried out in SKMC, Abu Dhabi.

The extracted RNA was transported in dry ice to the Khalifa University Center for Biotechnology (KU-BTC), Abu Dhabi along with all vital metadata information for each patient such as age, travel history, gender, clinical severity along with their signed consent forms.

The samples were subjected to q-RT PCR prior to viral genome sequencing using MIC-PCR system and Genesig Real-Time PCR Coronavirus COVID-19 (CE IVD) kit for measurement of viral RNA load in each sample. Samples with Cq-values less than 20 were selected for shot gun metagenome sequencing using TruSeq Stranded Total RNA Library Prep Kit with Ribo-Zero Gold (Cat. No. RS-122-2301) from Illumina (San Diego, CA, USA) on the NextSeq 500 platform at KU-BTC.

### SARS-CoV-2 shot gun metagenome sequencing: RNA-Seq Library preparation

Libraries were prepared using the Illumina TruSeq Stranded Total RNA Ribo Zero Gold (Catalog # 20020598) kit according to the manufacturer’s protocol with recommended RNA concentration of 200 ng/ul as the starting input for the protocol.

The methodology involved in the kit procedure was designed for initial depletion of ribosomal RNA and clean up, followed by fragmentation, cDNA synthesis (using the SuperScript II Reverse Transcriptase Kit from Invitrogen, Carlsbad, USA), adenylation, ligation of indexed adapters (Illumina TruSeq RNA UD Indexes–Catalog # 20022371), and amplification. The constructed libraries were amplified using 15 cycles of PCR.

Final libraries were quantified using DeNovix (DS-11 FX) and the library quality with size distribution was checked on Agilent Fragment Analyzer (M5310AA). All libraries were within an average size range of 260 to 380 bp, which were further normalized, pooled and diluted according to the Illumina NextSeq System Denature and Dilute Libraries Guide (15048776) for sequencing and loaded on the NextSeq 500 platform (San Diego, CA, USA) using the Illumina SP Reagent kit (300 cycles).

For quality control, two independent sequencing runs were performed. The first run involved sequencing the 37 investigated samples. The second run included duplicates of 78% of the samples (29 out of the 37). Specifically, the 29 duplicates were extracted from a split aliquot of the original sample received. These duplicate samples were sequenced separately (i.e., in run 2). The second sequencing run also included 3 negative controls (nuclease-free water). All three negative controls were free of contamination.

### Bioinformatics analysis

All 37 FASTQ files were subjected to trimming using Trimmomatic [24]. All samples were subjected to quality control using FastQC [25]. We subsequently performed reference-based genome assembly using BWA version 0.7.12 [26] against the SARS-CoV-2 reference strain Wuhan/Hu-1 (Genbank MN908947). We ensured high coverage throughout the reference genome using Qualimap [27] by retaining all strains with at least 95% of at least 10X -fold coverage of the reference genome. Variants are were called from on the resulting BAM files using Sam Tools/Picard 2.13.1 and GATK version 4.0.6.0 [28], performing the following steps: Sort Sam, Merge Sam Files, Mark Duplicates, Build BamIndex from the Picard suite; GATK’s HaplotypeCaller in discovery mode with ploidy set to 1, and finally we ran bcftools (version 1.10.2) consensus to generate VCF files using Haplotype Caller from the FASTA files. The workflow and the tools used in each step are provided in the S1 Fig. All FASTA files for samples that passed quality control were collated into one file.

### Global contextualization of SARS-CoV-2 experience

For global contextualization, we acquired 53,708 SARS-CoV-2 sequences from GISAID, as per 06/25/2020, in correspondence with the sampling period of UAE sequences. We filtered GISAID sequence records by various quality criteria: a sequence was retained, if the length was between 29,000 and 30,000 sequences, if the sampling date in the metadata was of recognizable format (including day and month) and from 2019 or 2020. We constructed a multiple sequence alignment for all local and all global GISAID sequences using augur’s default method mafft [29]. The N-terminus and C-terminus regions of many submitted sequences were either lacking, incomplete or of low quality, so all sequences were trimmed by 65 and 75 base pairs on the ends, respectively, and replaced with ‘N’, so as to maintain the reading frame for correct amino acid translation. We also removed sequences if the number of unknown nucleotides (N) exceeded 30 after clipping. This procedure yielded 28,412 high quality sequences. We then reduced redundancy by retaining only single representatives per country and sequence, yielding 17,044 sequences (script nr.py). We calculated a 17,044x69 distance matrix from the aligned sequences custom script (distances.py), which helps to identify the most similar sequences during the k-Nearest-Neighbor sequence selection: a custom script (preprocess.py) identifies a selection *σ* as the union of all global sequences that are similar to local sequences from the UAE:

$$\sigma =\underset{S\in UAE}{\cup }knn(s,GISAID)$$

where *knn* is the k-Nearest Neighbor function, providing for a UAE sample the k = 25 closest samples in the non-redundant GISAID dataset in terms of nucleotide differences. This form of subsampling makes sure that we did not miss highly relevant sequences (in particular, for the detection of international virus introductions), as could happen with pure random subsampling. E.g., if a specific variant evolves uniquely in country A, including a number of distinct mutations, and later a descendant spreads to country B, the connection between A and B can be lost due to random subsampling.

The selection of sequence neighbors was further complemented by random sampling to a total of 3,965 sequences (69 local and 3,894 global), though not including more than 100 sequences per country. The rationale behind this is that augur’s time resolution method TreeTime can also estimate probabilities for the geographic origin of ancestral nodes, but this process is very sensitive to sampling bias, and GISAID data is indeed very imbalanced with respect to country wide submission. While we focused on simple distinction between international and domestic transmissions, we argue that country-based downsampling mitigates GISAID’s sample imbalance. We used augur tree with default method IQtree [30] for the subsampled tree. A few very divergent international outliers were also removed from the tree. The augur refine method generates a calendar time-resolved phylogeny under the coalescent model, taking sampling dates into account (using TreeTime). It roots the tree on the reference strain (MN908947). TreeTime computes phylodynamic analysis using Maximum Likelihood, which has been shown to perform comparably with other methods used in the phylodynamic analysis of SARS-CoV-2 [7, 11].

Nextstrain/Augur generates a richly annotated phylogeny (augur refine), with ancestral nodes holding information on time and location, including an estimate of confidence for both. The phylogeny is visualized using Auspice (https://nextstrain.github.io/auspice/), which provides a rich set of exploration options. Figs 1, 2, and S2 Fig were generated using Auspice. The exact parameter settings are provided in nextstrainUAE.sh.

### Analysis of international and domestic transmissions

The annotated phylogeny was subjected to a recursive top-down algorithm that determines whether transmissions happened domestically or internationally. When parsing the phylogeny recursively, the origin of the ancestor was extracted from the augur annotation of the internal node. This information was passed on to the recursive calls to the subtrees. For each node (internal or leaf), a transmission was recorded as international or domestic, if the direct ancestor was of different origin than the current node or not, respectively. Estimates of the number of international/domestic transmissions were then calculated by averaging using a boxcar kernel (sliding window) with a width of 14 days. The code for this procedure is found in transmissions.py on the shared GitHub repository (https://github.com/Henschellab/GenEpidemiology).

### The UAE COVID-19 Collaborative Partnership

Juan Acuna, Eman Alefishat, Ernesto Damiani, Samuel F. Feng, Andreas Henschel, Abdulrahim Sajini, Ahmed Yousef (Khalifa University of Science and Technology, Abu Dhabi, United Arab Emirates); Bassam Ali (United Arab Emirates University, Al Ain, United Arab Emirates); Hiba Alhumaidan, Hala Imambabaccus, Amirtharaj Francis, Stefan Weber (Sheikh Khalifa Medical City and SEHA, Abu Dhabi, United Arab Emirates); Mohammad Tahseen Al Bataineh, Rabih Halwani, Rifat Akram Hamoudi (University of Sharjah, Sharjah, United Arab Emirates); Abdulmajeed Al Khajeh, Laila Salameh (Dubai Health Authority, Dubai, United Arab Emirates) for the COVID-19 Collaborative Partnership lead by Habiba S Alsafar (Khalifa University of Science and Technology, Abu Dhabi, United Arab Emirates), E-mail: habiba.alsafar@ku.ac.ae.

## Supporting information

S1 Table Study sample demographic characteristics. (PDF)

S2 Table Global PANGOLIN lineage assignment of the study’s SARS-COV-2 genomes. (PDF)

S3 Table Observed amino acid mutations of particular interest. The listed mutations are either of high entropy, frequency or (mostly) unique to UAE. Abbreviations: Prev–prevalence, e–entropy, AD–Abu Dhabi.
(PDF)

S4 Table Intra-host Single Nucleotide Variants with MAF > 5%. Abbreviations: Pos—position (in the reference genome), Ref–reference nucleotide, Alt–Alternative nucleotide, D_Ref_, D_Alt_—allelic depth of reference and alternative nucleotide, respectively, MAF%—minor allele frequency in percent.
(DOCX)

S1 Fig Sequencing and analysis pipeline. (TIF)

S2 Fig UAE clades from Fig 1 with MRCA from UAE with confidence between 66% and 99%. The four clades (Clade 1 (A), Clade 4 (B), Clade 8 (C) and Clade 6 (D), include samples collected from Dubai and Abu Dhabi, indicating local transmissions.
(TIF)

S3 Fig Global comparison of international (blue) and domestic (orange) transmissions over time. The y-axis holds the total number of cases as a 14-day moving average, with respect to the samples chosen for phylogeny construction (). The dotted and dash-dotted vertical lines (green) mark the time of the travel ban to/from the countries and the earliest minimum number of departures during travel ban, respectively. International (blue) and domestic (orange) transmissions.
(ZIP)
