# Supplementary material for: Travel ban effects on SARS-CoV-2 transmission lineages in the UAE as inferred by genomic epidemiology
Source: PLoS One. 2022 Mar 2;17(3):e0264682. doi: 10.1371/journal.pone.0264682 (PMC8890736; doi:10.1371/journal.pone.0264682)
Supplement: S1 Table — (PDF) [file pone.0264682.s001.pdf]

S1 Table. Study sample demographic characteristics.

Sample ID	Age	Gender	Nationality	Sampling Date	Collection City	GISAID Accession
UAE/H29	8	Female	UAE	03/04/2020	Abu Dhabi	EPI_ISL_528538
UAE/H18	40	Male	Pakistan	24/04/2020	Abu Dhabi	EPI_ISL_528713
UAE/H8	50	Male	Bangladesh	24/04/2020	Abu Dhabi	EPI_ISL_528720
UAE/H9	33	Male	Egypt	24/04/2020	Abu Dhabi	EPI_ISL_528721
UAE/H21	46	Male	India	25/04/2020	Abu Dhabi	EPI_ISL_528716
UAE/H10	55	Male	Syria	25/04/2020	Abu Dhabi	EPI_ISL_528710
UAE/H5	42	Male	Egypt	25/04/2020	Abu Dhabi	EPI_ISL_528719
UAE/H13	28	Male	Pakistan	26/04/2020	Abu Dhabi	EPI_ISL_528712
UAE/H20	3	Male	Egypt	26/04/2020	Abu Dhabi	EPI_ISL_528715
UAE/H23	31	Male	UAE	26/04/2020	Abu Dhabi	EPI_ISL_528717
UAE/H2	24	Male	Nepal	26/04/2020	Abu Dhabi	EPI_ISL_528714
UAE/H12	30	Male	India	26/04/2020	Abu Dhabi	EPI_ISL_528711
UAE/12B	59	Male	Egypt	28/04/2020	Abu Dhabi	EPI_ISL_528686
UAE/14B	30	Male	Nepal	28/04/2020	Abu Dhabi	EPI_ISL_528688
UAE/16B	25	Male	India	28/04/2020	Abu Dhabi	EPI_ISL_528690
UAE/31B	45	Male	India	29/04/2020	Abu Dhabi	EPI_ISL_528697
UAE/H1	29	Female	Pakistan	30/04/2020	Abu Dhabi	EPI_ISL_528709
UAE/H27	25	Female	India	30/04/2020	Abu Dhabi	EPI_ISL_528718
UAE/2	30	Female	India	02/05/2020	Abu Dhabi	EPI_ISL_528692
UAE/16	44	Female	UAE	05/05/2020	Abu Dhabi	EPI_ISL_528689
UAE/13	45	Male	India	05/05/2020	Abu Dhabi	EPI_ISL_528687
UAE/21R	34	Male	Bangladesh	05/05/2020	Abu Dhabi	EPI_ISL_528693
UAE/44	48	Male	India	11/05/2020	Abu Dhabi	EPI_ISL_528701
UAE/42	37	Male	India	12/05/2020	Abu Dhabi	EPI_ISL_528699
UAE/38	43	Male	India	12/05/2020	Abu Dhabi	EPI_ISL_528698
UAE/56	26	Female	Philippine	14/05/2020	Abu Dhabi	EPI_ISL_528706
UAE/54	31	Male	Egypt	14/05/2020	Abu Dhabi	EPI_ISL_528704
UAE/306	29	Male	India	02/06/2020	Dubai	EPI_ISL_528694
UAE/310	31	Male	India	02/06/2020	Dubai	EPI_ISL_528695
UAE/313	38	Male	India	02/06/2020	Dubai	EPI_ISL_528696
UAE/C2-109	65	Male	UAE	03/06/2020	Abu Dhabi	EPI_ISL_528708
UAE/195	71	Male	Egypt	07/06/2020	Abu Dhabi	EPI_ISL_528691
UAE/434	61	Male	Sudan	13/06/2020	Abu Dhabi	EPI_ISL_528700
UAE/484	33	Female	Philippine	15/06/2020	Dubai	EPI_ISL_528702
UAE/529	39	Male	UAE	26/06/2020	Dubai	EPI_ISL_528703
UAE/558	44	Female	UAE	30/06/2020	Abu Dhabi	EPI_ISL_528705
UAE/581	24	Female	UAE	01/07/2020	Abu Dhabi	EPI_ISL_528707
