# Supplementary material for: Travel ban effects on SARS-CoV-2 transmission lineages in the UAE as inferred by genomic epidemiology
Source: PLoS One. 2022 Mar 2;17(3):e0264682. doi: 10.1371/journal.pone.0264682 (PMC8890736; doi:10.1371/journal.pone.0264682)
Supplement: S2 Table — (PDF) [file pone.0264682.s002.pdf]

## S2 Table. Global PANGOLIN lineage assignment of the study's SARS-COV-2 genomes

| Lineage | Samples |
|---|---|
| **A** | UAE/H12, UAE/H29, UAE/306 |
| **B.1** | UAE/31B, UAE/21R, UAE/H2, UAE/H20, UAE/H8, UAE/558, UAE/581 |
| **B.1.1** | UAE/12B, UAE/14B, UAE/16B, UAE/13, UAE/16, UAE/2, UAE/42, UAE/44, UAE/54, UAE/56, UAE/H1, UAE/H10, UAE/H13, UAE/H18, UAE/H21, UAE/H23, UAE/H27, UAE/H9, UAE/195, UAE/484, UAE/38, UAE/38, UAE/529, UAE/310, UAE/434 |
| **B.1.1.7** | UAE/313 |
| **B.1.5** | UAE/H5 |
