# Supplementary material for: Travel ban effects on SARS-CoV-2 transmission lineages in the UAE as inferred by genomic epidemiology
Source: PLoS One. 2022 Mar 2;17(3):e0264682. doi: 10.1371/journal.pone.0264682 (PMC8890736; doi:10.1371/journal.pone.0264682)
Supplement: S3 Table — The listed mutations are either of high entropy, frequency or (mostly) unique to UAE. Abbreviations: Prev–prevalence, e–entropy, AD–Abu Dhabi. (PDF) [file pone.0264682.s003.pdf]

S3 Table. Observed amino acid mutations of particular interest. The listed mutations are either of high entropy, frequency or (mostly) unique to UAE.

Protein	Mutation	Nucleotide substitution	Prev AD	e_{AD}	Prev UAE	e_{UAE}	Prev World	e_{World}	Uniqueness
ORF1a	V1887I	G5924A	11	0.609	11	0.55	11	0.019	unique
ORF1b	P314L	C14408T	33	0.343	46	0.637	high	0.576	common
ORF14	G50N	G28881A, G28882A, G28883C	24	0.63	26	0.68	high	0.614	common
S	E583D	G23311C	4	0.343	4	0.221	6	0.013	4/6 cases in UAE only
S	D614G	A23403G	34	0.343	50	0.6337	high	0.574	common
S	Q613H	G23401T	1	0.124	1	0.105	5	0.01	Only in UAE and Japan

Prev – prevalence, e – entropy, AD – Abu Dhabi.
