# Supplementary material for: Travel ban effects on SARS-CoV-2 transmission lineages in the UAE as inferred by genomic epidemiology
Source: PLoS One. 2022 Mar 2;17(3):e0264682. doi: 10.1371/journal.pone.0264682 (PMC8890736; doi:10.1371/journal.pone.0264682)
Supplement: S4 Table — Abbreviations: Pos—position (in the reference genome), Ref–reference nucleotide, Alt–Alternative nucleotide, DRef, DAlt—allelic depth of reference and alternative nucleotide, respectively, MAF%—minor allele frequency in percent. (DOCX) [file pone.0264682.s004.docx]

**Table S4**. **Intra-host Single Nucleotide Variants with MAF > 5%.**

|  |  | | | | | **Rerun H18** | | | |
| --- | --- | --- | --- | --- | --- | --- | --- | --- | --- |
| **Sample** | **Locus** | **D_ref_** | **D_alt_** | **MAF (%)** | **Depth** | **D_ref2_** | **D_alt2_** | **MAF(%)** | **Depth_2_** |
| H18 | 241 | 2 | 33 | 6.06 | 35 | 166 | 925 | 17.95 | 1091 |
| H18 | 3037 | 2 | 14 | 14.29 | 16 | 167 | 1079 | 15.48 | 1246 |
| H18 | 5924 | 18 | 73 | 24.66 | 91 | 122 | 944 | 12.92 | 1066 |
| H18 | 14408 | 7 | 28 | 25.00 | 35 | 196 | 1048 | 18.70 | 1244 |
| H18 | 21775 | 0 | 16 | 0.00 | 16 | 191 | 829 | 23.04 | 1020 |
| H18 | 23403 | 2 | 30 | 6.67 | 32 | 161 | 1066 | 15.10 | 1227 |
| H18 | 28881 | 14 | 33 | 42.42 | 47 | 198 | 748 | 26.47 | 946 |
| H18 | 28882 | 16 | 32 | 50.00 | 48 | 197 | 748 | 26.34 | 945 |
| H18 | 28883 | 16 | 34 | 47.06 | 50 | 195 | 748 | 26.07 | 943 |
| H5 | 7945 | 35 | 165 | 21.21 | 200 |  |  |  |  |
| 16B | 11083 | 11 | 106 | 10.38 | 117 |  |  |  |  |
| 16B | 12102 | 40 | 82 | 48.78 | 122 |  |  |  |  |

All calls are made with GATK Genotype quality 99 (maximum). Pos - position (in the reference genome), D_Ref_, D_Alt_ - allelic Depth of reference and alternative nucleotide, respectively, and MAF % - minor allele frequency percentage.
