# Supplementary figures and images for: Travel ban effects on SARS-CoV-2 transmission lineages in the UAE as inferred by genomic epidemiology
Source: PLoS One. 2022 Mar 2;17(3):e0264682. doi: 10.1371/journal.pone.0264682 (PMC8890736; doi:10.1371/journal.pone.0264682)

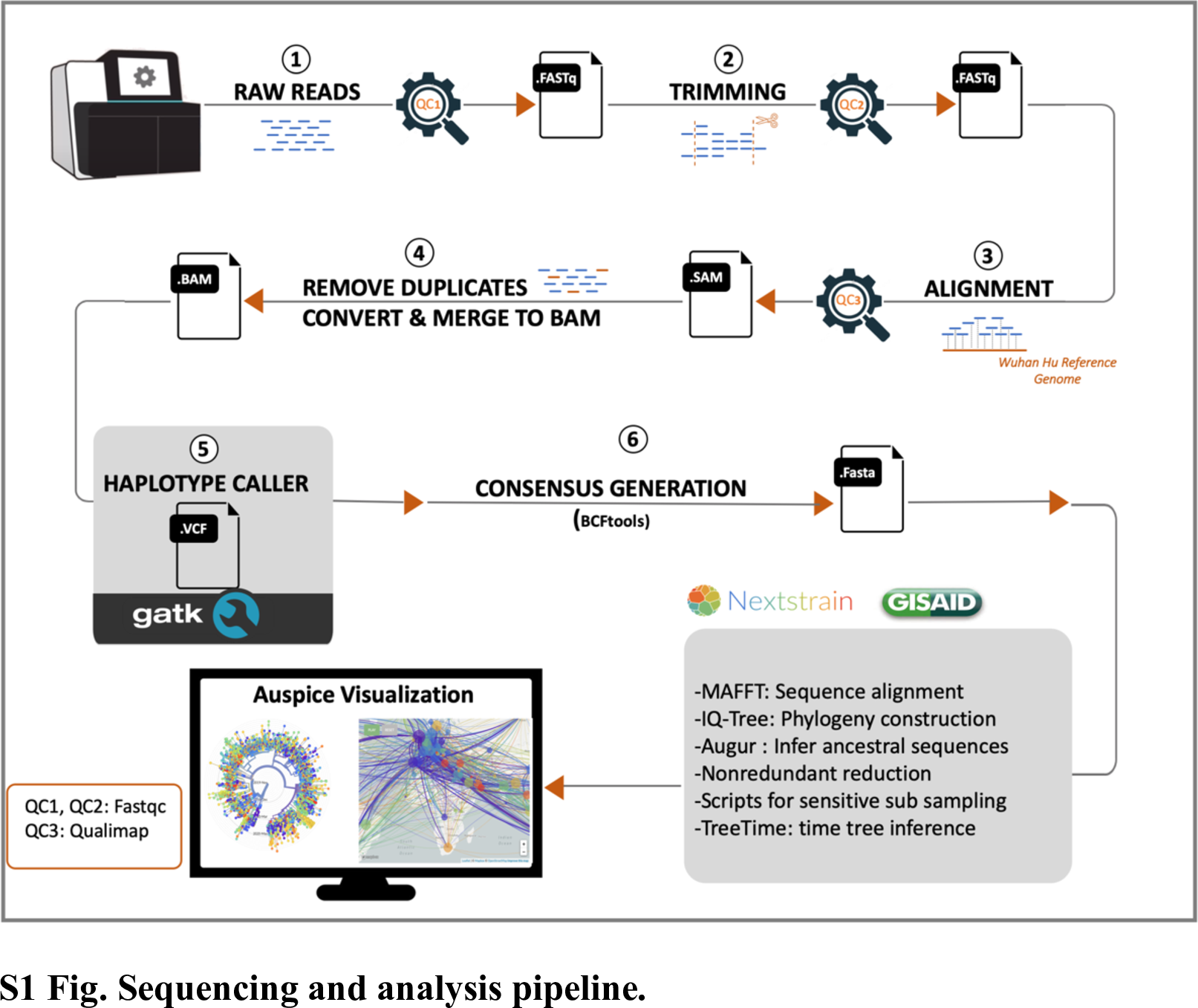

Supplement: S1 Fig — (TIF) [file pone.0264682.s005.tif]

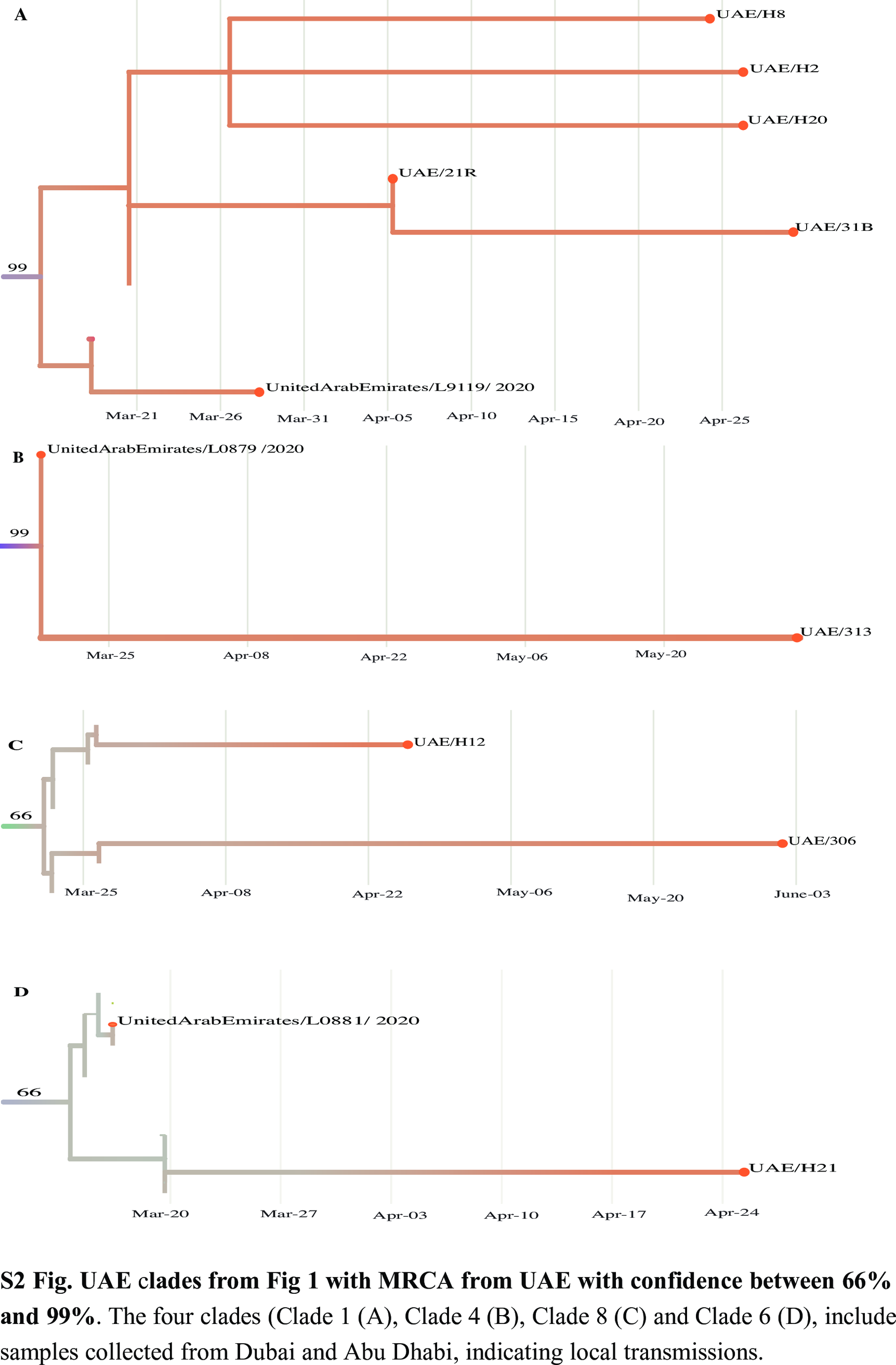

Supplement: S2 Fig — The four clades (Clade 1 (A), Clade 4 (B), Clade 8 (C) and Clade 6 (D), include samples collected from Dubai and Abu Dhabi, indicating local transmissions. (TIF) [file pone.0264682.s006.tif]

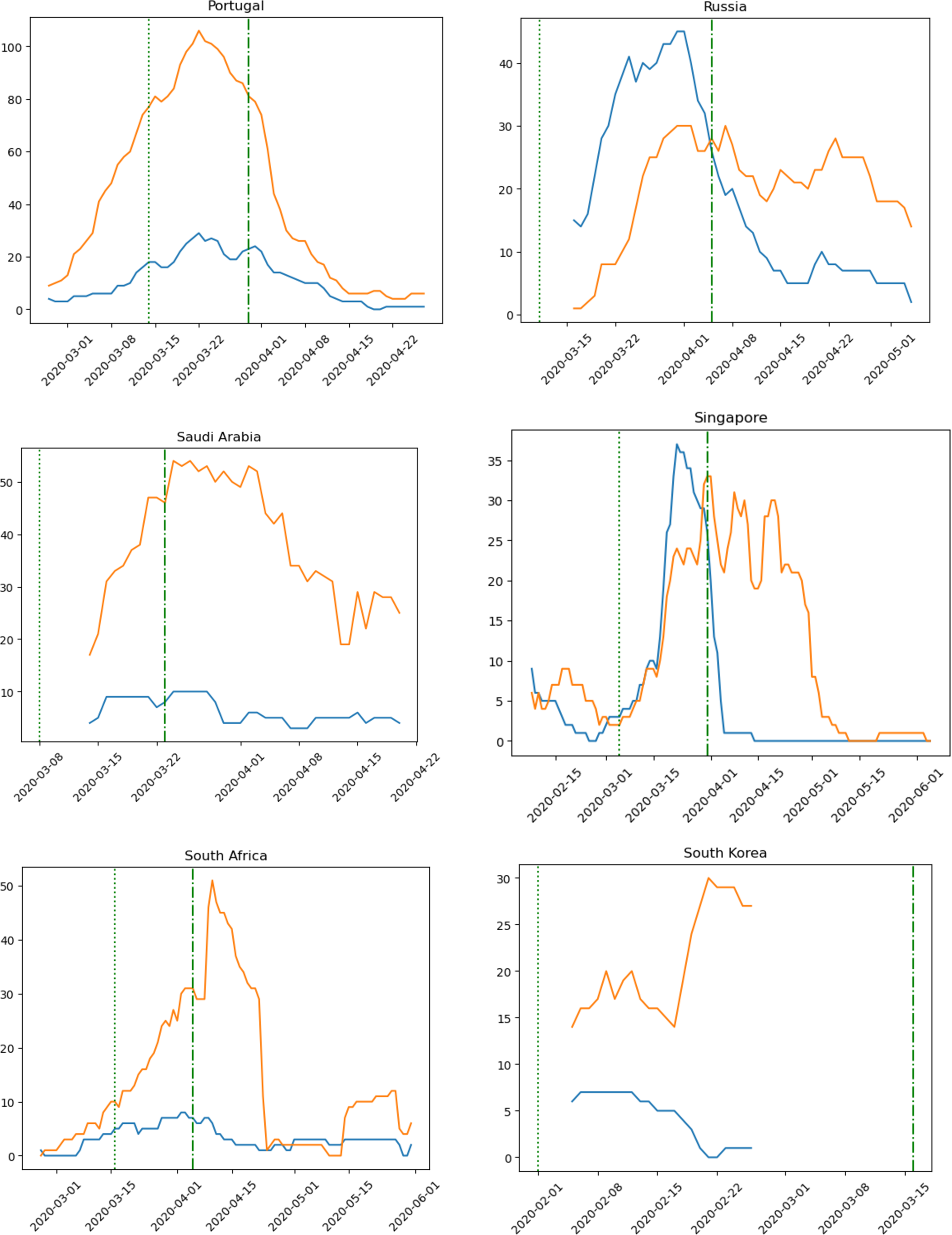

Supplement: S3 Fig — The y-axis holds the total number of cases as a 14-day moving average, with respect to the samples chosen for phylogeny construction (). The dotted and dash-dotted vertical lines (green) mark the time of the travel ban to/from the countries and the earliest minimum number of departures during travel ban, respectively. International (blue) and domestic (orange) transmissions. (ZIP) [file pone.0264682.s007.zip › S3 Fig-4.tif]

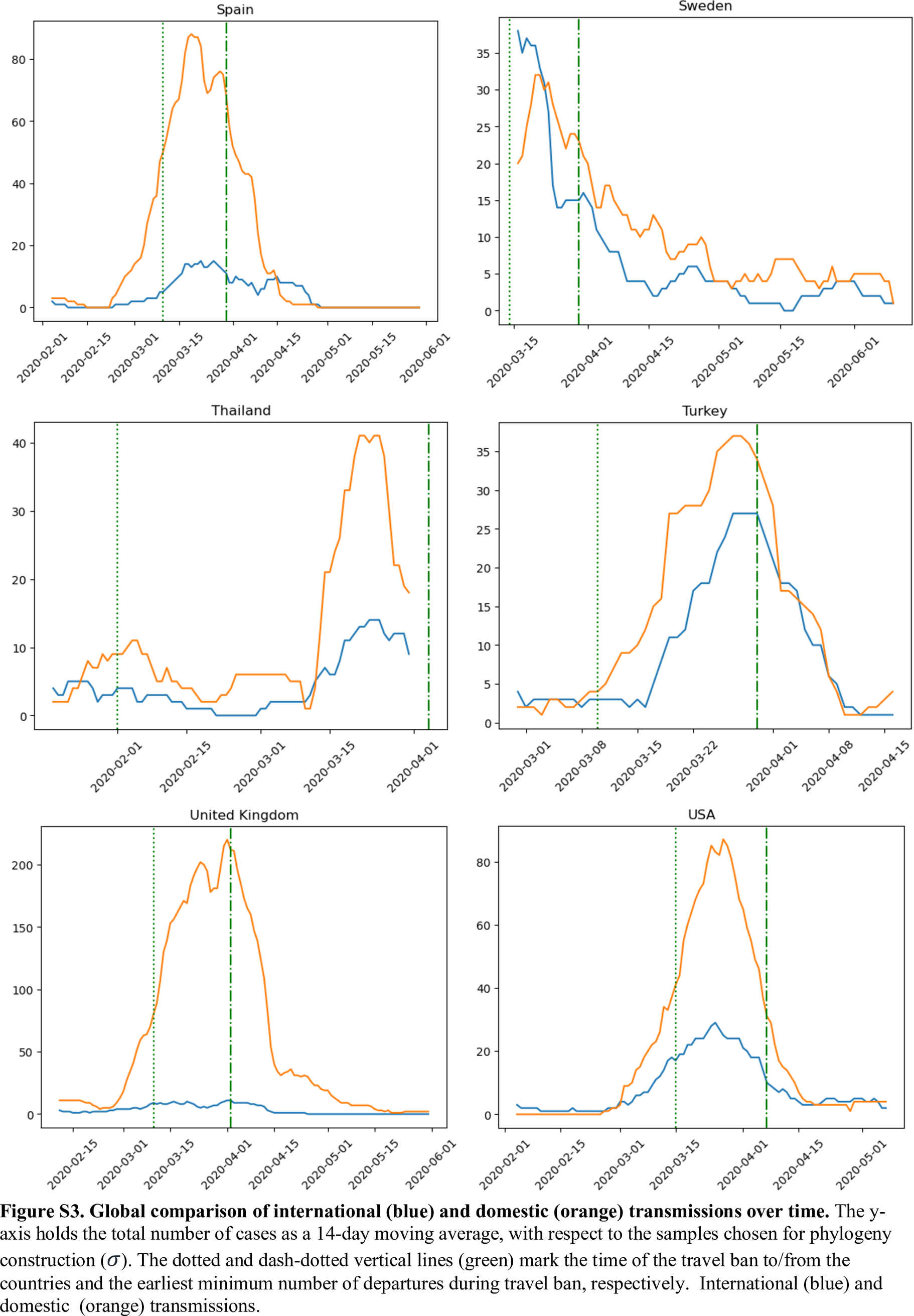

Supplement: S3 Fig — The y-axis holds the total number of cases as a 14-day moving average, with respect to the samples chosen for phylogeny construction (). The dotted and dash-dotted vertical lines (green) mark the time of the travel ban to/from the countries and the earliest minimum number of departures during travel ban, respectively. International (blue) and domestic (orange) transmissions. (ZIP) [file pone.0264682.s007.zip › S3 Fig-5.tif]

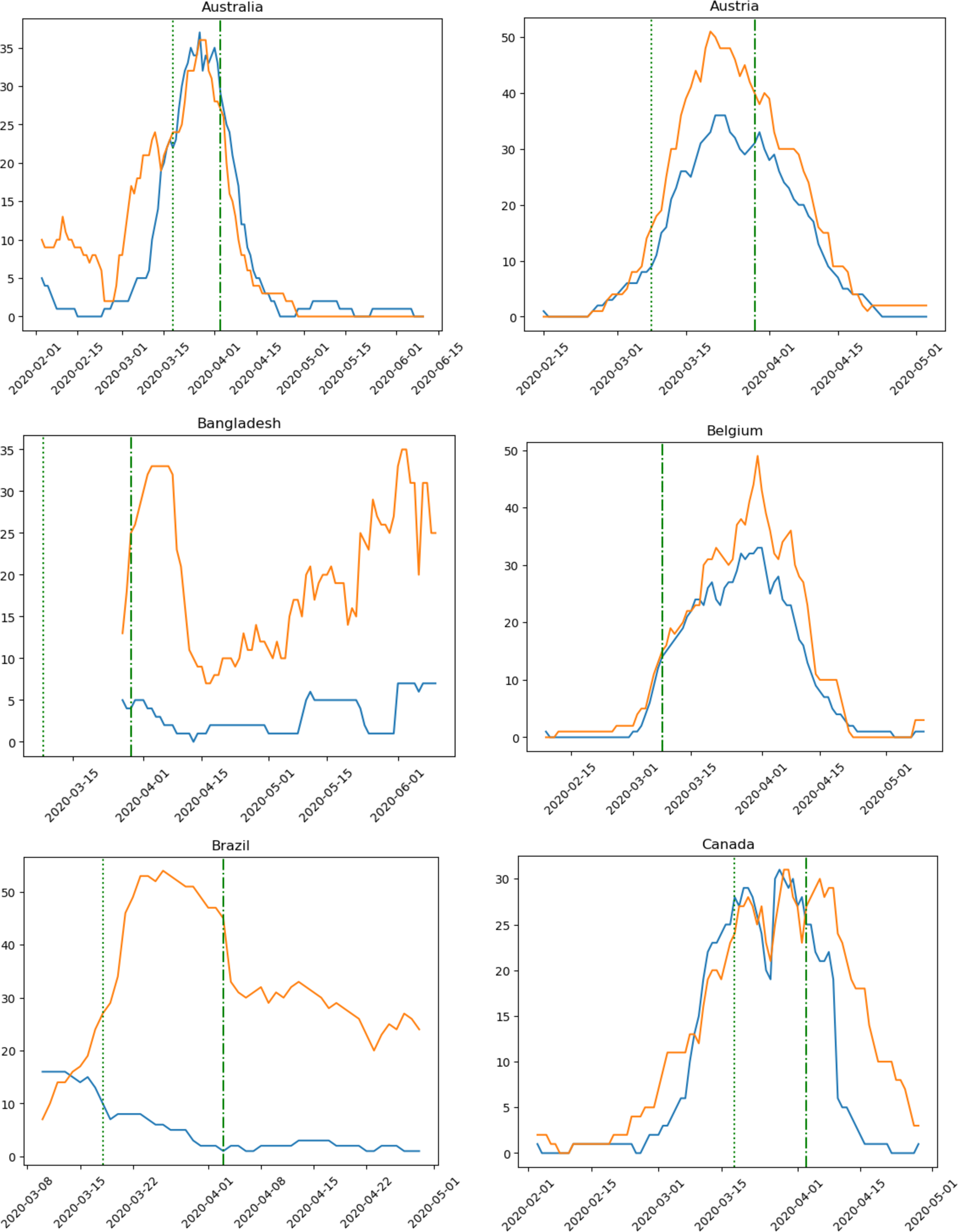

Supplement: S3 Fig — The y-axis holds the total number of cases as a 14-day moving average, with respect to the samples chosen for phylogeny construction (). The dotted and dash-dotted vertical lines (green) mark the time of the travel ban to/from the countries and the earliest minimum number of departures during travel ban, respectively. International (blue) and domestic (orange) transmissions. (ZIP) [file pone.0264682.s007.zip › S3 Fig-1.tif]

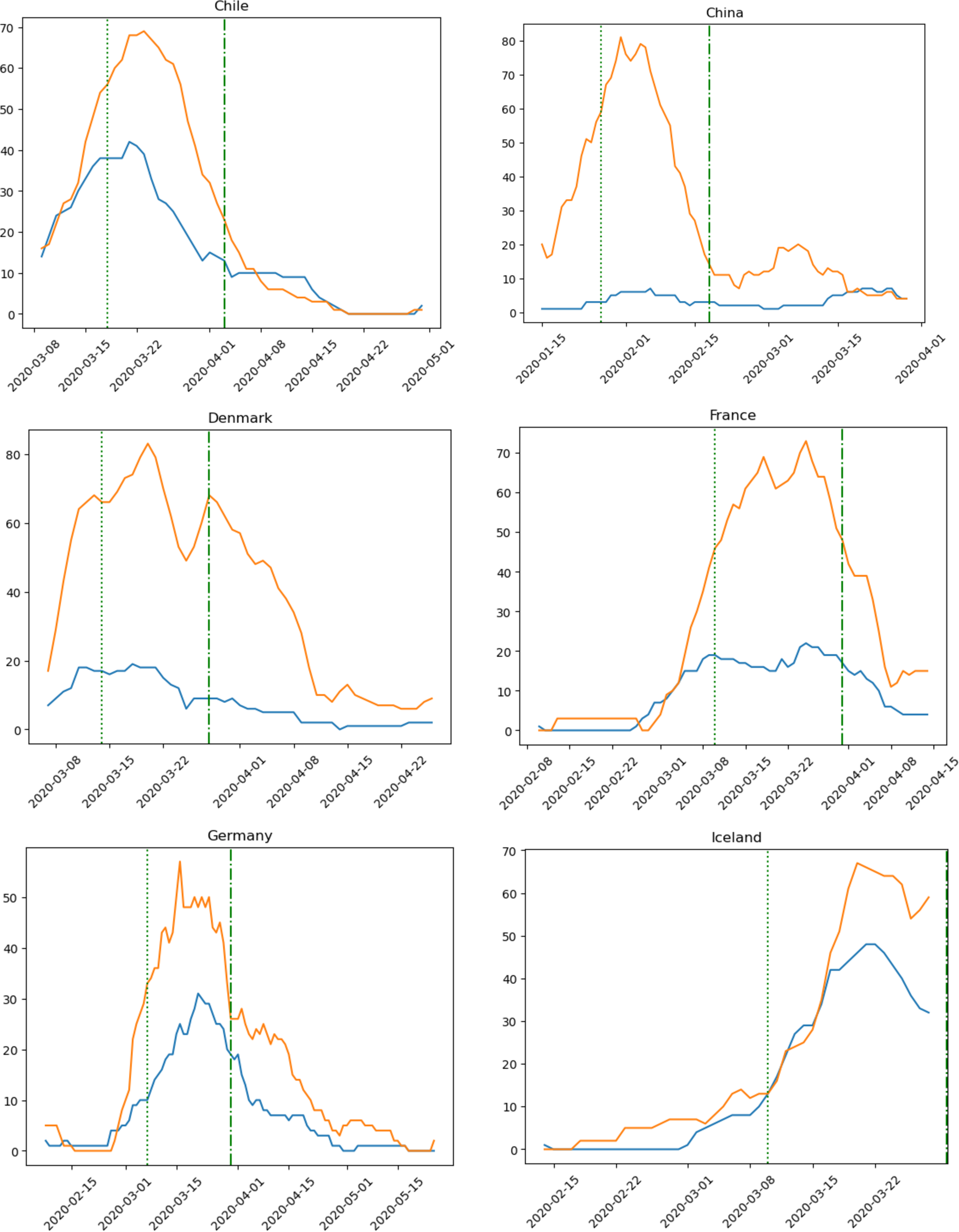

Supplement: S3 Fig — The y-axis holds the total number of cases as a 14-day moving average, with respect to the samples chosen for phylogeny construction (). The dotted and dash-dotted vertical lines (green) mark the time of the travel ban to/from the countries and the earliest minimum number of departures during travel ban, respectively. International (blue) and domestic (orange) transmissions. (ZIP) [file pone.0264682.s007.zip › S3 Fig-2.tif]

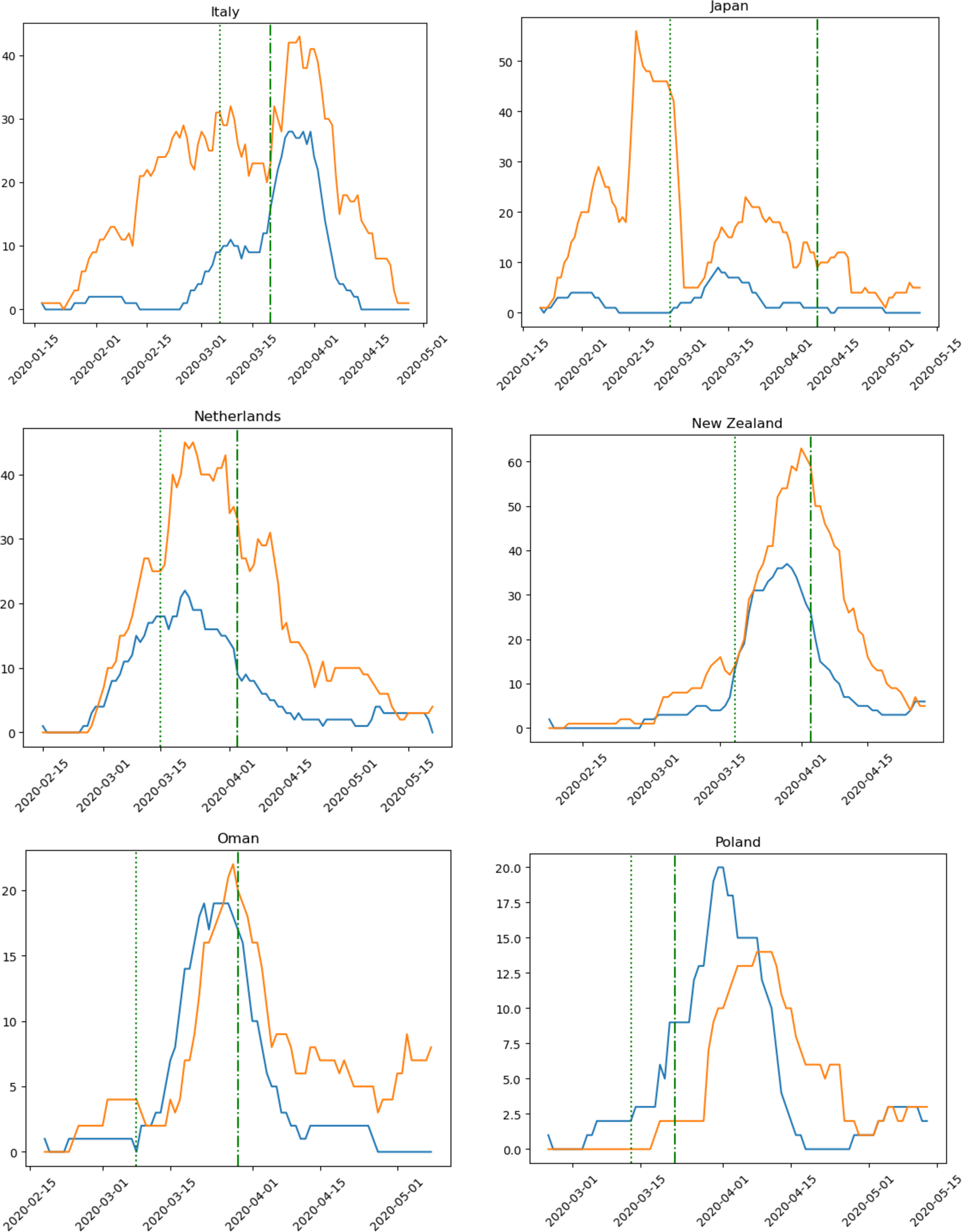

Supplement: S3 Fig — The y-axis holds the total number of cases as a 14-day moving average, with respect to the samples chosen for phylogeny construction (). The dotted and dash-dotted vertical lines (green) mark the time of the travel ban to/from the countries and the earliest minimum number of departures during travel ban, respectively. International (blue) and domestic (orange) transmissions. (ZIP) [file pone.0264682.s007.zip › S3 Fig-3.tif]
